# Supplementary material for: Recruitment Kinetics of XRCC1 and RNF8 Following MeV Proton and α-Particle Micro-Irradiation
Source: Biology (Basel). 2023 Jun 27;12(7):921. doi: 10.3390/biology12070921 (PMC10376363; doi:10.3390/biology12070921)
Supplement: Supplementary file 1 [file biology-12-00921-s001.zip › biology-2405939-supplementary.pdf]

Recruitment Kinetics of XRCC1 and RNF8 Following MeV Proton and α-Particle Micro-Irradiation

Giovanna Muggiolu[1], Eva Torfeh[1], Marina Simon[1], Guillaume Devès[1], Hervé Seznec[1] and Philippe Barberet[1]

[1]University Bordeaux, CNRS, LP2I, UMR 5797, 33170 Gradignan, France

Supplementary Data

Table S1. GFP-XRCC1 kinetics after proton and ⊚-particle micro-irradiations. The variations of T_2 parameter allowed to separate the cells behavior in two main groups: in the first one (a) the fluorescence intensity decreases after reaching the maximum value; in the second one (b) the fluorescence intensity does not decrease. The recruitment time is not influenced by these variations.

Ion	Ions per Point/ Nucleus	Dose (Gy)	Number of Cells Analyzed	Intensity of Fluorescence ± SD	Mean Recruitment Time (s) ± SD	Mean Decay Time (s) ± SD
**3 MeV ⚜**	10	1	8	0.4 ± 0.3	117 ± 93	3102 ± 179 (a)
**3 MeV ⚜**	10	1	8	0.3 ± 0.01	307 ± 187	1.0E+05 ± 9.7E+04 (b)
**3 MeV ⚜**	50	5	36	1.5 ± 0.4	259 ± 127	3678 ± 1819
**3 MeV ⚜**	100	10	29	1.5 ± 0.7	160 ± 29	4200 ± 2507
**3 MeV ⚜**	1000	100	34	3.8 ± 1.4	34 ± 14	5343 ± 2155
**3 MeV H+**	100	1	28	0.4 ± 0.2	233 ± 83	5336 ± 7639 (a)
**3 MeV H+**	100	1	18	0.5 ± 0.3	315 ± 113	1.7E+05 ± 5.2E+05 (b)
**3 MeV H+**	500	5	28	1.9 ± 1.1	221 ± 142	1697 ± 2300 (a)
**3 MeV H+**	500	5	10	1.2 ± 0.5	210 ± 51	2.0E+05 ± 2.9E+05 (b)
**3 MeV H+**	1000	10	25	2.4 ± 0.7	138 ± 44	11120 ± 28607

Table S2. GFP-RNF8 kinetics after proton and ⊚-particle irradiations.

Ion	Ions per Point/Nucleus	Dose (Gy)	Number of Cells Analyzed	Mean Recruitment Time (s) ± SD
**3 MeV ⚜**	1	0.1	28	452 ± 210
**3 MeV ⚜**	10	1	27	524 ± 254
**3 MeV ⚜**	100	10	26	223 ± 105
**3 MeV H+**	100	1	31	755 ± 445
**3 MeV H+**	1000	10	16	937 ± 425
